# Laparoendoscopic single‐site simple nephrectomy and reduced port procedure for inflammatory nonfunctioning kidney

**DOI:** 10.1002/iju5.12278

**Published:** 2021-03-17

**Authors:** Go Kaneko, Seiya Hattori, Suguru Shirotake, Kent Kanao, Satoshi Hara, Masafumi Oyama

**Affiliations:** ^1^ Department of Uro‐Oncology Saitama Medical University International Medical Center Hidaka Saitama Japan; ^2^ Department of Urology Kawasaki Municipal Hospital Kawasaki Kanagawa Japan

**Keywords:** inflammatory nonfunctioning kidney, laparoscopy, LESS, simple nephrectomy, reduced port surgery

## Abstract

**Introduction:**

To describe laparoendoscopic single‐site simple nephrectomy and reduced port simple nephrectomy for inflammatory nonfunctioning kidney.

**Case presentation:**

Case 1: a 58‐year‐old female with fever was referred to our hospital. Computed tomography demonstrated a markedly atrophic right kidney and mild hydronephrosis. Case 2: a 64‐year‐old male with a history of several intra‐abdominal surgeries visited our hospital with a complaint of left back pain and fever. Computed tomography demonstrated left marked hydronephrosis, thinning of renal parenchyma, and duplicated inferior vena cava. After antibiotic treatment, transperitoneal reduced port simple nephrectomy and retroperitoneal laparoendoscopic single‐site simple nephrectomy were performed in Case 1 and 2, respectively, because the function of the affected kidney was almost lost on renography. Although adhesion was slightly noted around the renal hilum in Case 1, neither conversion to laparotomy nor placement of additional ports was needed.

**Conclusion:**

Laparoendoscopic single‐site simple nephrectomy and reduced port simple nephrectomy for inflammatory nonfunctioning kidney may be options for experienced laparoscopic surgeons.

**Abbreviations & Acronyms**
CT: computed tomography
INFK: inflammatory nonfunctioning kidney
IVC: inferior vena cava
LESS‐SN: laparoendoscopic single‐site simple nephrectomy
LSN: laparoscopic simple nephrectomy
RP‐SN: reduced port simple nephrectomy

**Keynote message**

LESS‐SN and RP‐SN for INFK may be options for experienced laparoscopic surgeons due to a high level of cosmesis.

## Introduction

LSN for inflammatory renal conditions has been considered contraindication because of its higher rates of complications.^1^ However, due to advances in surgical devices and more reports of LSN for inflammatory kidney, it is no longer contraindicated.^2^, ^3^, ^4^

Laparoendoscopic single‐site nephrectomy and reduced port laparoscopic nephrectomy were recently introduced,^5^, ^6^ and their feasibility, safety, and better cosmetic outcomes were reported. However, the details of LESS‐SN and RP‐SN for inflammatory kidney have not been reported. As their feasibility and safety are unclear, detailed descriptions are useful for laparoscopic surgeons. We describe our experience of LESS‐SN and RP‐SN for INFK with detailed figures and videos.

## Case presentation

### Case 1

**Fig. 1:**
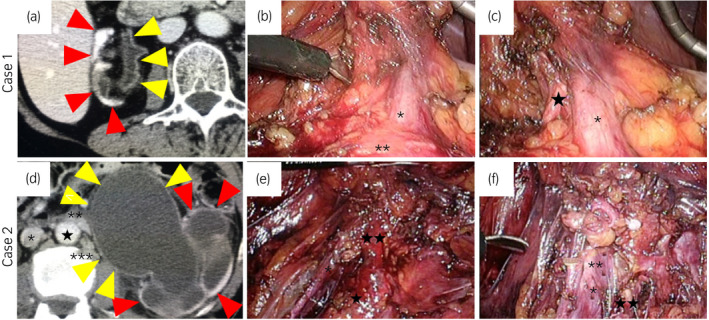
(a) CT demonstrated a markedly atrophic right kidney and mild hydronephrosis in Case 1. Red and yellow arrows show the right kidney and mild hydronephrosis, respectively. (b,c) Intraoperative findings of the renal hilum in Case 1 (*: right renal vein, **: IVC, ★: right renal artery). (d) CT demonstrated left marked hydronephrosis and a duplicated IVC in Case 2. Red and yellow arrows show the left kidney and marked hydronephrosis, respectively (*: right IVC, **: left renal vein, ***: left IVC, ★: aorta). (e,f) Intraoperative findings of the renal hilum in Case 2 (*: left IVC, **: left renal vein, ★: aorta, ★★: left renal artery). The renal artery is divided in f.

A 58‐year‐old female (body mass index 17.7 kg/m^2^) with no medical history was referred to our hospital with recurring fever every 2 weeks for 6 months. Her body temperature was 38.3℃ and right costovertebral angle tenderness was detected. Marked pyuria was found in urinary sediment (white blood cells ≥100/high power field). On hematological examination, the inflammatory response was slightly risen (white blood cells 9200/μL, C‐reactive protein 1.84 mg/dL). CT demonstrated a markedly atrophic right kidney and mild hydronephrosis (Fig. 1a). She was initially treated by antibiotics, and her symptoms resolved, and inflammatory markers in the blood examination reached normal ranges. However, right renal function was almost abolished based on renography and right transperitoneal RP‐SN was planned.

### Case 2

A 64‐year‐old man (body mass index 21.2 kg/m^2^) with a history of surgery for appendicitis, ileus, and abdominal incision hernia visited our hospital because of left back pain and fever. On hematological examination, inflammatory markers were markedly high (white blood cells 18 200/μL and C‐reactive protein 27.53 mg/dL). CT demonstrated left marked hydronephrosis, thinning of renal parenchyma and duplicated IVC (Fig. 1d). The left IVC directly drained into the left renal vein just anterior to the left renal artery. He was initially treated by antibiotics and inflammatory markers gradually decreased. As left renal function was abolished on renography, left retroperitoneal LESS‐SN was planned.

### Surgical technique

#### Case 1

Transperitoneal RP‐SN was performed the same as conventional LSN. The patient was placed in a left lateral position and a multichannel port (SILS^™^ port; Covidien, Tokyo, Japan) was inserted through an umbilical zigzag skin incision.^7^ A bent laparoscopic instrument (SILS^™^ Clinch; Covidien), a straight laparoscopic electrode (Opti 4; Covidien), a straight vessel sealing device (Enseal; Ethicon, Tokyo, Japan), and a 5‐mm flexible laparoscope (Olympus Surgical, Tokyo, Japan) were used. A 5‐mm trocar was additionally inserted at the anterior axillary line caudal to the arcus costalis to lift the liver. Adhesions were slightly found only around the renal artery (Fig. 1b); however, it was possible to carefully dissect it from the surrounding tissue (Fig. 1c). After the renal artery and vein were divided, the kidney was dissected from the surrounding tissue.

#### Case 2

Retroperitoneal LESS‐SN was performed the same as conventional retroperitoneoscopic simple nephrectomy. The patient was placed in a right lateral position with slight flexion. The retroperitoneum was dilated by a balloon dissector through a 2.5‐cm skin incision between the tip of the 12th rib and anterior superior iliac spine, and a multichannel port (SILS^™^ port; Covidien) was inserted. A bent laparoscopic instrument, a straight laparoscopic electrode, straight vessel sealing device (LigaSure^™^ Maryland; Covidien), and a 5‐mm flexible laparoscope were used. The left IVC was present along the aorta (Fig. 1e) and directly drained into the left renal vein just anterior to the left renal artery (Fig. 1f). No adhesions were found around the kidney. Both the renal artery and vein were divided without difficulty, and the kidney was dissected from the surrounding tissue.

Detailed intraoperative findings in Cases 1 and 2 are shown in Videos S1 and S2, respectively.

### Operative results

**Fig. 2:**
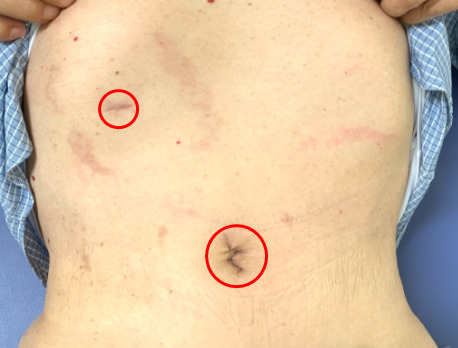
The surgical wound at 1 month after surgery.

The pneumoperitoneum time and estimated blood loss in Case 1 and 2 were 108 and 103 min, and 3 and 5 mL, respectively. In both cases, the postoperative course was uneventful and the histopathological diagnosis was chronic pyelonephritis. The surgical wound at 1 month after surgery in Case 1 is shown in Figure 2, and it was inconspicuous at 3 months after surgery.

## Discussion

We described our experience of LESS‐SN and RP‐SN for INFK. All procedures were safely performed in both cases and cosmetic outcomes were excellent. Furthermore, LESS‐SN was able to be safely performed even in the case with major vascular anomaly.

LSN for INFK is now one of the options for experienced laparoscopic surgeons because of its minimal invasiveness. LESS or reduced port surgery for several diseases has been gradually spreading in the urological field.^7^, ^8^, ^9^, ^10^, ^11^, ^12^, ^13^, ^14^, ^15^ However, detailed descriptions of LESS‐SN and RP‐SN for INFK have not been reported. Therefore, we described our experience of LESS‐SN or RP‐SN for INFK with detailed figures and videos. Although their minimal invasiveness is attractive, a guarantee of safety is the most important point. The accumulation of surgical experience is needed for wider use of LESS‐SN and RP‐SN for INFK.

We selected a transperitoneal and retroperitoneal approach in Case 1 and 2, respectively. We usually prefer the transperitoneal approach in LSN because it has the advantages of a wide surgical field and high manipulation area. However, the major vascular anomaly of a duplicated IVC was detected on preoperative imaging studies in Case 2. As described above, the left IVC directly drained into the left renal vein just anterior to the renal artery; therefore, difficulty in finding the left renal artery via a transperitoneal approach was of concern. In addition, the patient had a history of several intra‐abdominal surgeries; therefore, we selected a retroperitoneal approach. The renal hilum and large vessels were promptly and accurately recognized without difficulty, and the renal hilum was able to be safely handled. The surgical approach should be flexibly selected based on the physician’s preference and patient factors, such as anatomy and past surgical history, for LESS‐SN and RP‐SN.

We used an umbilical zigzag skin incision in Case 1 and a cosmesis was excellent. An umbilical zigzag skin incision increases the diameter of the fascial and peritoneal opening to 6 cm; therefore, a larger sized wound protector can be inserted.^16^ As interference between the forceps and the laparoscope is unlikely, bent instruments may no longer be necessary in LESS‐SN or RP‐SN if a larger wound protector is inserted from an umbilical zigzag skin incision for INFK. This reduction of technical difficulty may aid in the spread of these procedures. However, the distance between ports of SILS^™^, which was used in Case 1, is short; therefore, a bent laparoscopic instrument was required to prevent interference between the forceps and the laparoscope. We are planning on trying LESS‐SN and RP‐SN for INFK using a larger wound protector and only straight laparoscopic instruments from an umbilical zigzag skin incision.

## Conclusion

LESS‐SN and RP‐SN for INFK were safely performed, and cosmetic outcomes were excellent. LESS‐SN and RP‐SN for INFK may be an option for experienced laparoscopic surgeons.

## Conflict of interest

The authors declare no conflict of interest.

## Supporting information


**Video S1.** Intraoperative findings of reduced port laparoscopic simple right nephrectomy via a transperitoneal approach.

**Video S2.** Intraoperative findings of laparoendoscopic single‐site simple left nephrectomy via a retroperitoneal approach.
